# Age-Specific Differences in Oncogenic Pathway Deregulation Seen in Human Breast Tumors

**DOI:** 10.1371/journal.pone.0001373

**Published:** 2008-01-02

**Authors:** Carey K. Anders, Chaitanya R. Acharya, David S. Hsu, Gloria Broadwater, Katherine Garman, John A. Foekens, Yi Zhang, Yixin Wang, Kelly Marcom, Jeffrey R. Marks, Sayan Mukherjee, Joseph R. Nevins, Kimberly L. Blackwell, Anil Potti

**Affiliations:** 1 Division of Medical Oncology, Department of Medicine, Duke University, Durham, North Carolina, United States of America; 2 Institute for Genome Sciences and Policy, Duke University, Durham, North Carolina, United States of America; 3 Cancer Center Biostatistics, Duke University Medical Center, Durham, North Carolina, United States of America; 4 Erasmus Medical Center, Rotterdam, The Netherlands; 5 Veridex, Inc., Johnson and Johnson, San Diego, Californa, United States of America; National Cancer Institute at Frederick, United States of America

## Abstract

**Purpose:**

To define the biology driving the aggressive nature of breast cancer arising in young women.

**Experimental Design:**

Among 784 patients with early stage breast cancer, using prospectively-defined, age-specific cohorts (young ≤45 years; older ≥65 years), 411 eligible patients (n = 200≤45 years; n = 211≥65 years) with clinically-annotated Affymetrix microarray data were identified. GSEA, signatures of oncogenic pathway deregulation and predictors of chemotherapy sensitivity were evaluated within the two age-defined cohorts.

**Results:**

In comparing deregulation of oncogenic pathways between age groups, a higher probability of PI3K (p = 0.006) and Myc (p = 0.03) pathway deregulation was observed in breast tumors arising in younger women. When evaluating unique patterns of pathway deregulation, a low probability of Src and E2F deregulation in tumors of younger women, concurrent with a higher probability of PI3K, Myc, and β-catenin, conferred a worse prognosis (HR = 4.15). In contrast, a higher probability of Src and E2F pathway activation in tumors of older women, with concurrent low probability of PI3K, Myc and β-catenin deregulation, was associated with poorer outcome (HR = 2.7). In multivariate analyses, genomic clusters of pathway deregulation illustrate prognostic value.

**Conclusion:**

Results demonstrate that breast cancer arising in young women represents a distinct biologic entity characterized by unique patterns of deregulated signaling pathways that are prognostic, independent of currently available clinico-pathologic variables. These results should enable refinement of targeted treatment strategies in this clinically challenging situation.

## Introduction

Young women diagnosed with breast cancer have a poorer overall survival and are twice as likely to recur when compared with older counterparts [1]–[3]. Many investigations have addressed the basis for the aggressive nature of breast cancer arising in young women. Multiple hypotheses exist, including the lower prevalence of hormone receptor positivity, higher grade tumors, larger tumors, and a higher incidence of Her2 overexpression, lymphovascular invasion, and lymph node positive disease among young women [2], [4], [5]. Despite a higher incidence of negative prognostic factors, young age as a single variable has consistently proven to be an independent predictor of adverse outcome [6]–[8]. At the present time, the underlying biology driving the aggressive nature of breast cancer arising in young women has yet to be defined.

We recently reported that gene-expression signatures can be identified to reflect the status of several important oncogenic pathways (i.e. Ras, Myc, E2F, β-catenin, and Src) that are central to both cell growth and fate [9]. We have evaluated the clinical significance of patterns of oncogenic pathway deregulation in over 700 patients with primary breast tumors to illustrate the unique biologic phenotype of breast cancer arising in young women.

## Materials and Methods

### Dataset and Patient Selection

Four, publicly-available datasets were employed to perform our analysis: Bild et al. (GSE3143) [9], Wang et al. (GSE2034) [10], Ivshina et al. (GSE4922) [11], and a large cohort of samples from the Duke tumor bank (GEO accession user name: ander118, password: IGSP). The selected datasets, based on either the Affymetrix Human Genome U133A or U95 array, provided clinically-annotated gene expression probabilities from early stage breast tumors. For details specific to each dataset, see Supplementary Table S1 [12]. Prior to applying signatures of pathway deregulation, all data was RMA-normalized. Further, for U95 data, a previously described annotation and cross reference tool (Chipcomparer) that facilitates for comparison across Affymetrix platforms (U95 and U133) by matching corresponding probeids and relative expression values was employed. The complete details are described in the Supplementary Methods S1 [9], [13]. In total, 784 clinically-annotated breast tumor samples were available for analysis. All samples were analyzed and reported according to MIAME guidelines.

Initially, we prospectively, pre-defined tumors arising in young versus older women by performing a receiver operator characteristics (ROC) curve based on estrogen receptor (ER) status and patient age at the time of breast cancer diagnosis based on the historical observation that breast tumors arising in younger women characteristically exhibit quantitatively less ER expression when compared to older women (Supplementary Figure S1) [4]. Based on the results of the ROC curve, young age was defined as ≤45 years (p<0.001) and data from 200 women met this age cut-off. Tumors arising in women ≥65 years (n = 211) were selected to represent an older, post-menopausal comparison group. The remaining 373 patients were between the ages of 45 and 65 years and were not included in this analysis as our goal was to compare breast tumors arising at the extremes of age.

### Oncogenic Pathway and Chemotherapy Sensitivity Analyses

Previously described signatures of oncogenic pathway deregulation and chemotherapy sensitivity were applied to clinically-annotated microarray data using MatLab Software, Version7.0.4 as detailed in Supplementary Methods S1 [9], [13]–[16].

In brief, oncogenic pathway signatures were developed using human primary mammary epithelial cell cultures and recombinant adenoviruses expressing various oncogenic activities in an otherwise quiescent cell. RNA from multiple independent transfections was collected for DNA microarray analysis using the Affymetrix Human Genome U133 Plus 2.0 Array. Gene expression signatures reflecting the activity of a given oncogenic pathway were identified defining a relevant phenotype-related metagene. Regression models then assigned the relative probability of pathway deregulation in tumor or cell line samples [9]. A correlation was observed between the likelihood of pathway deregulation and biochemical and molecular correlates, including mutational analyses of the individual genes involved in the pathway (i.e. Ras) [9].

Gene expression signatures predicting sensitivity to individual chemotherapeutic drugs were developed using the NCI-60 panel from the National Cancer Institute (NCI) as described previously [13]. Briefly, genes correlating most highly with drug sensitivity were identified and a Bayesian binary regression analysis differentiated a pattern of drug sensitivity from that of resistance. A gene expression signature was then identified classifying cell lines and tumors on the basis of chemotherapy sensitivity.

Specific to this analysis, heatmaps generated via hierarchical clustering were generated using “R” software (http://www.r-project.org/). Complete linkage clustering was performed using an open source development software project, bioconductor, ver 1.9, for the analysis of microarray expression data with the uncentered correlation similarity metric.

Standard Kaplan-Meier survival curves were generated for clusters of patients with similar patterns of oncogenic pathway deregulation using GraphPad Prism Software, version 4.03. Differences in survival were tested for statistical significance using a two-sided log-rank test using GraphPad Prism Software, version 4.03. This test generates a two-tailed P value testing the null hypothesis, which is that the survival curves are identical in the overall populations. Therefore, the null hypothesis is that the populations have no differences in disease-free survival.

Individual differences in the probability of oncogenic pathway deregulation between women aged ≤45 years and ≥65 years were analyzed via the non-parametric Mann-Whitney U test using Graph Pad Prism Software, version 4.03. A two-sided p-value less than 0.05 was considered statistically significant. Results were validated via Gene Set Enrichment Analysis (GSEA) methodology (http://www.broad.mit.edu/gsea/). GSEA is a computational method that determines whether an a priori defined set of genes shows statistically significant, concordant differences between two biological states [17].

Finally, chemotherapy sensitivity patterns were assessed across groups of patients defined by both pathway clusters and age (≤45 years and ≥65 years). Linear regression analyses were performed to ascertain correlations where indicated, using Graph Pad Prism Software, version 4.03.

### Multivariate Analyses

Our goal was to determine if cluster (of oncogenic pathway deregulation) designation was independently significant when controlling for known clinico-pathologic variables in the prediction of disease-free survival in early stage breast cancer. A disease-free survival event was defined as the time from diagnosis to recurrence or death, whichever occurred first, and was censored at time of last follow-up for those who were alive. Multivariate Cox Proportional Hazards regression modeling was used to predict disease-free survival when considering each age cohort separately. The clinico-pathologic variables considered included: age at breast cancer diagnosis, ER and progesterone receptor (PR) status by immunohistochemistry or enzyme immunoassay (IHC or EIA, positive vs. negative), Her2 by IHC (0, 1+, 2+ vs. 3+), tumor grade (1, 2 vs. 3), tumor size (≤2 cm vs. >2 cm), and lymph node status (positive vs. negative). The dataset with clinico-pathologic variables was excellent for imputing missing values due to high correlations among the variables. For multivariate modeling SAS proc MI was used to impute missing clinico-pathologic data for variables with less than 50% missing values. For this reason, PR and Her2 were excluded from all multivariate models. Multivariate models used the backward selection technique and an alpha of 0.50 [18].

## Results

### Clinical Characteristics

Clinico-pathologic and demographic data including age at diagnosis, ER status, lymph node status, and tumor size, with corresponding gene expression data were available for patients in all four datasets. PR and Her2 status were available only in the Duke and CODEX datasets, respectively. A detailed description of the clinico-pathologic and demographic information for all patients included in the study is shown in Supplementary Table S2 [12].

### Signatures of Oncogenic Pathway Deregulation in Young Women

The power of gene expression profiling is the ability to understand biology beyond what may be apparent from the study of clinical variables or individual gene markers. Nevertheless, the interpretation of large-scale expression data can be a significant challenge. We have described an alternative approach that makes use of expression signatures of oncogenic signaling pathways that can be used to profile the status of oncogenic pathways in a collection of biological samples, including human tumors.

**Figure 1:**
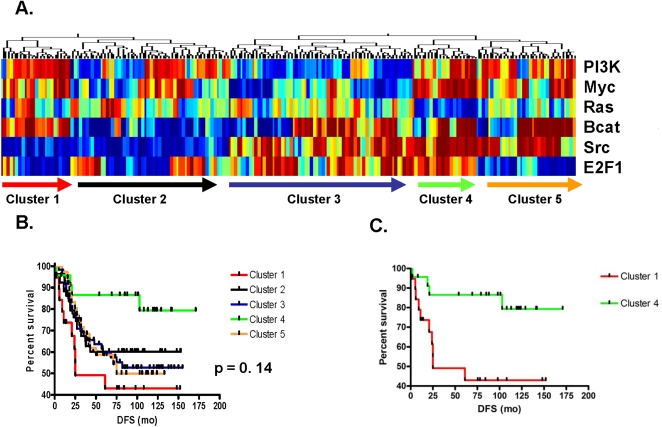
Patterns of pathway deregulation in human breast tumors arising in women aged ≤45 years. A) Prediction of PI3K, Myc, Ras, β-catenin, Src and E2F pathway deregulation. Red represents high probability of pathway deregulation, blue represents low probability of pathway deregulation. Five clusters emerge based on pathway patterns. B) Kaplan Meier survival analysis based on pathway patterns: good prognosis (cluster 4), intermediate prognosis (clusters 2, 3, 5), and poor prognosis (cluster 1), (p = 0.14). C) Kaplan Meier survival analysis comparing clinically-significant differences between clusters 1 and 4 (HR 4.15).

Using the previously described signatures of oncogenic pathway deregulation, patterns of pathway deregulation in 200 breast tumor samples arising in young women aged ≤45 years were evaluated. Hierarchical clustering revealed clear patterns of oncogenic pathway deregulation defining five main clusters (Figure 1A). Analysis of disease-free survival of patients identified by these clusters revealed clinically-significant distinctions as a function of the pattern. Patients in cluster 4 exhibited very good prognosis, patients in three clusters illustrated intermediate prognosis (clusters 2, 3, and 5) and patients in cluster 1 illustrated a very poor prognosis (p = 0.14) (Figure 1B). In further exploration of subgroups, patients defined by cluster 1 had a poorer disease-free survival when compared to those defined by cluster 4 (HR 4.15) (Figure 1C).

In evaluating patterns of pathway deregulation between patients with extremes in prognosis (Cluster 1 and 4), the most striking difference is that of Src and E2F. Patients in the poorest prognosis cluster (cluster 1) are characterized by a low probability of Src and E2F deregulation in the setting of high probability of PI3K, Myc and β-catenin deregulation. Conversely, patients in the good prognosis cluster (cluster 4) are characterized by high probability of Src and E2F deregulation again in the setting of a high probability of PI3K, Myc and β-catenin deregulation. The differential expression of Src and E2F sheds light on the biology and subsequent behavior of breast cancer arising in young women, but begs the question if these patterns of pathway deregulation are governed by other important prognostic variables such as hormone receptor status.

Breast tumors arising in younger women are known to express lower levels of ER [4]. To clarify whether or not the pathway analysis of young women's tumors is influenced by ER status, we stratified young women by ER status and re-evaluated oncogenic pathway deregulation and correlated findings with disease-free survival. Among young women with ER-positive breast tumors, two main clusters emerged, again driven by the Src pathway. Interestingly, the same pattern was seen among ER-negative breast tumors arising in young women (Supplementary Figure S2A and S2B, left panels). In analyzing the disease free survival between clusters, however, the prognosis is similar for both ER-positive and ER-negative tumors (p = 0.34 and p = 0.32, respectively) (Supplementary Figure S2A and S2B, right panels). Given the comparable patterns of pathway activation and corresponding prognosis despite ER classification, we conclude that the patterns of oncogenic pathway deregulation among breast tumors arising in young women in this analysis are independent of ER status. This was further confirmed in a multivariate analysis of clinico-pathologic variables and genomic clusters of pathway deregulation. This analysis illustrated that among women aged ≤45 years, younger age at diagnosis (HR 2.22, p<0.001) was the most significant predictor of inferior outcome. Although not statistically significant, larger tumor size (HR 1.39, p = 0.19) and positive lymph node status (HR 1.24, p = 0.38) were correlated with a poorer prognosis. Importantly, genomic pathway cluster designation remained within the model as an important predictor of clinical outcome (cluster 1 vs. cluster 4; HR 4.31, p = 0.18) (Table 1).

**Table 1:** Multivariate Analysis Among Women Aged ≤45 years

Variable	Hazard Ratio	p-value
Age, younger	2.22	<0.001
Tumor size, >2cm	1.39	0.19
Lymph node, positive	1.24	0.38
Cluster 1 vs 4	4.31	0.18*

* 4 degrees of freedom test

Further, we elected to evaluate oncogenic pathway deregulation among tumors arising in young women as a function of additional clinico-pathologic variables. Stratification of young women's tumors by independent characteristics, including nodal status, Her2, tumor grade and size, did not reveal a statistically significant difference in prognosis between clusters defined by pathway deregulation (Supplementary Figures S3, S4, S5 and S6), further supporting the conclusion that patterns of oncogenic pathway deregulation seen in young women are independent of clinico-pathologic features.

### Biologic Comparison of Age–specific Cohorts with Breast Cancer

**Figure 2:**
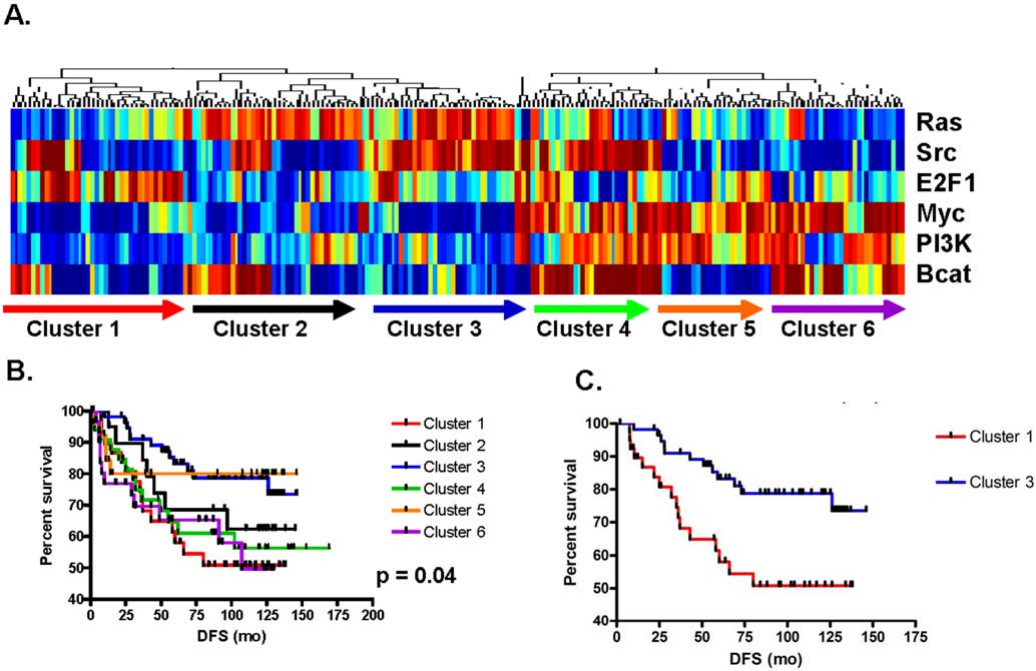
Patterns of pathway deregulation in human breast tumors arising in women ≥aged 65 years. A) Prediction of PI3K, Myc, Ras, β-catenin, Src and E2F pathway deregulation. Red represents high probability of pathway deregulation, blue represents low probability of pathway deregulation. Six clusters emerge based on pathway patterns. B) Kaplan Meier survival analysis for breast cancer patients aged ≥65 years based on pathway patterns: good prognosis (clusters 3,5) and poor prognosis (clusters 1,6), (p = 0.04). C) Kaplan Meier survival analysis for breast cancer patients comparing clinically-significant differences between clusters 1 and 3 (HR = 2.7).

In parallel with the evaluation of breast tumors arising in young women, an analysis of breast tumors arising in women aged ≥65 years was concurrently performed to provide a comparison group, allowing the results generated among younger women to be placed into context. Patterns of pathway deregulation in 211 breast tumor samples arising in women aged ≥65 years were evaluated. Six main clusters emerged of which two illustrate superior prognosis (cluster 3 and cluster 5) and two illustrates an inferior prognosis (cluster 1 and cluster 6) (p = 0.04) (Figure 2A and 2B). The most distinct difference in disease-free survival was illustrated between clusters 1 and 3 (HR = 2.7, Figure 2C). Poor prognosis tumors in cluster 1 are characterized by a high probability of Src and E2F deregulation with concurrent low probability of Myc, PI3K, and β-catenin deregulation. Conversely, good prognosis tumors in cluster 3 are characterized by a high probability of Src and Ras deregulation in the setting of a low probability of Myc, PI3K and β-catenin deregulation. These results suggest that the differential expression of Ras and E2F may be a driving force underlying the nature of breast cancer arising in older women.

**Figure 3:**
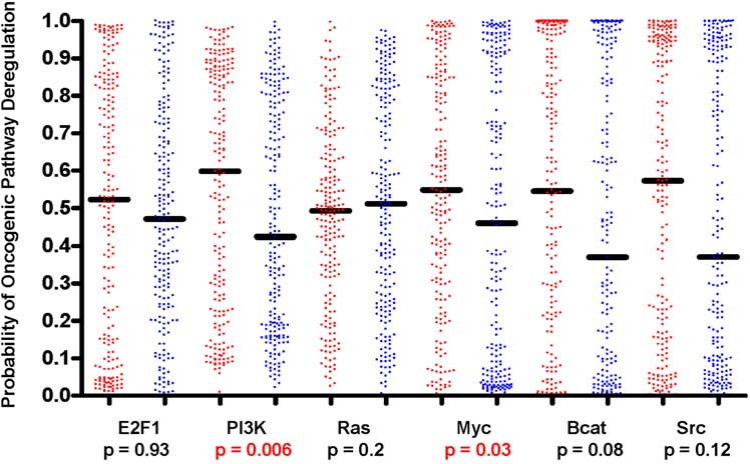
Non-parametric T test evaluating pathway probability between tumors arising in younger versus older women. Red represents women aged ≤45 years. Blue represents women aged ≥65 years. The line represents the median.

We also directly compared the probability of pathway deregulation between tumors arising in patients ≤45 years and ≥65 years of age. Pathways evaluated included E2F, PI3K, Ras, Myc, β-catenin and Src. There was a higher probability of PI3K and Myc pathway deregulation observed in tumors arising in younger women when compared to older women and this difference was statistically significant (Mann-Whitney U test, p = 0.006 and p = 0.03, respectively) (Figure 3). The fact that, when evaluated individually, only two oncogenic pathways were significantly different between tumors arising in younger versus older women speaks to the importance of evaluating patterns of oncogenic pathway deregulation to gain a deeper understanding of the contributing biologic processes working in concert.

Additionally, as a further validation of our observations, Gene Set Enrichment Analysis (GSEA) was employed to identify differences in gene expression profiles from tumors arising in women aged ≤45 years (class 1) and ≥65 years (class 2), thus validating the above findings [17]. Results confirm that gene sets involved in both the Myc (p = 0.008) and the AKT pathways (p = 0.029), a downstream effector of PI3K, were differentially expressed in tumors arising in younger women in contrast to their older counterparts [19].

Multivariate modeling evaluating classification of breast tumors arising in older women by oncogenic pathway clustering and important clinico-pathologic variables was additionally performed. Among older women, larger tumor size (HR 2.51, p<0.001) was the only significant predictor of disease free survival. Additionally, negative ER status (HR 1.47, p = 0.25), higher grade tumors (HR 1.62, p = 0.13), positive lymph node status (HR 1.66, p = 0.10) and genomic clusters of pathway deregulation (HR 2.22, p = 0.10) remained in the model suggesting prognostic values of these variables. (Table 2). In contrast to the analysis of young women, age was not a significant predictor of outcome among older women, again highlighting the profound influence of young age on breast cancer prognosis.

**Table 2:** Multivariate Analysis Among Women Aged ≥65 years

Variable	Hazard Ratio	p-value
Tumor size, >2cm	2.51	<0.001
Nuclear Grade, 3	1.62	0.13
ER status, negative	1.47	0.25
Lymph node, positive	1.66	0.10
Cluster 1 vs 3	2.22	0.10*

* 5 degrees of freedom test

### Chemotherapy Sensitivity Patterns

**Figure 4:**
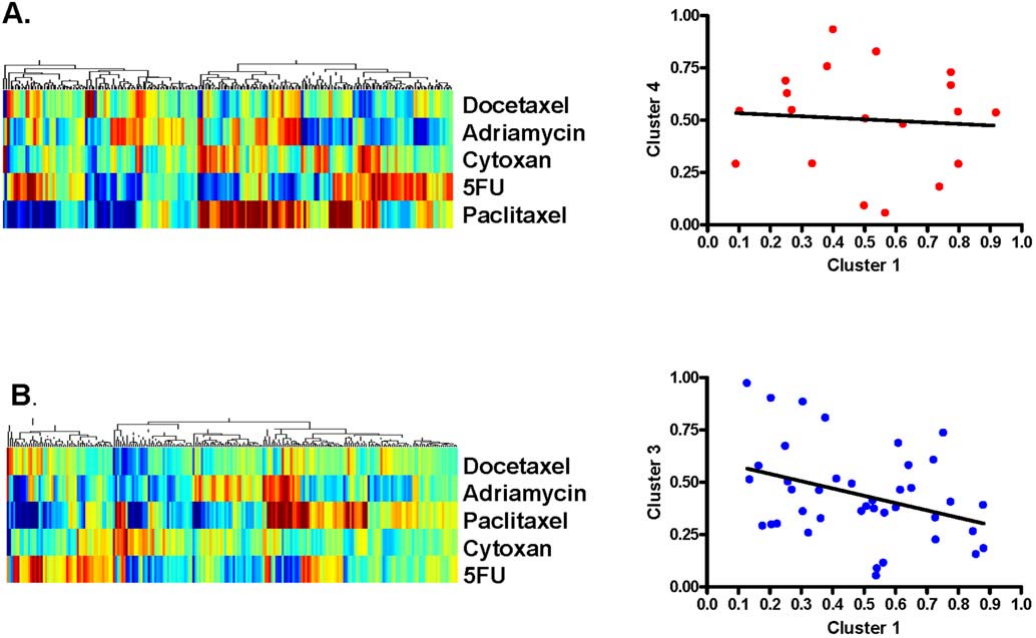
Chemosensitivity patterns among women aged ≤45 years and ≥65 years with early stage breast cancer. A) LEFT PANEL Hierarchical clustering of the probability of sensitivity to 5-fluorouracil (5-FU), paclitaxel, docetaxel, adriamycin and cyclophosphamide among 200 breast tumors arising in women aged ≤45 years; RIGHT PANEL Linear regression analysis of sensitivity to adriamycin among women in genomic cluster 1 (poor prognosis) vs. 4 (good prognosis) aged ≤45 years. B) LEFT PANEL Hierarchical clustering of the probability of sensitivity to 5FU, paclitaxel, docetaxel, adriamycin and cyclophosphamide among 211 breast tumors arising in women aged ≥65 years. RIGHT PANEL Linear regression analysis of sensitivity to adriamycin among women in genomic cluster 1 (poor prognosis) vs. genomic cluster 3 (good prognosis), demonstrating a statistically significant (p = 0.02, log rank) relationship between clusters (of pathway deregulation) and adriamycin sensitivity.

Finally, genomic-derived signatures of 5-fluorouracil (5-FU), paclitaxel, docetaxel, adriamycin and cyclophosphamide sensitivity were applied to identify unique patterns of chemotherapy sensitivity by age [12]. There was no statistically significant difference in sensitivity to 5FU, paclitaxel, docetaxel, adriamycin and cyclophosphamide between women aged ≤45 years or ≥65 years (data not shown). However, as shown in Figure 4A and 4B, left panels, distinct patterns of chemotherapy sensitivity exist between age groups.

As a proof-of-principle, we then utilized genomic clusters of pathway deregulation (as described in Figures 1 and 2) and evaluated the likelihood of sensitivity by cytotoxic agent. Among women aged ≤45 years, there was no statistically significant difference in predicted chemotherapeutic sensitivities to 5FU, paclitaxel, docetaxel, adriamycin or cyclophosphamide between patients in genomic cluster 1 (poor prognosis) or genomic cluster 4 (good prognosis) (Figure 4A, right panel). In contrast, among women aged ≥65 years, there was a statistically significant difference in predicted chemotherapeutic sensitivity to adriamycin between patients in genomic cluster 1 (poor prognosis, high probability of E2F deregulation) and genomic cluster 3 (good prognosis, high probability of Ras deregulation) (p = 0.02, log rank) (Figure 4B, right panel). Although predicting the likelihood of sensitivity to individual chemotherapeutic drugs will greatly advance the care of patients with cancer, our findings are hypothesis-generating and highlight the importance of developing a prognostic and predictive strategy to incorporate therapies, as guided by the results of oncogenic pathway deregulation, into the management of early stage breast cancer.

## Discussion

Breast cancer arising in young women is characterized by a higher incidence of negative prognostic factors, higher recurrence rates and poorer overall survival despite aggressive therapies [1]–[5]. Clearly, the poor survival of this group of patients emphasizes the importance of identifying molecular characteristics that might be exploited for new therapeutic strategies. To date, the underlying biology driving the aggressive nature of this disease entity has yet to be fully elucidated. More recently, gene expression profiles and oncogenic pathway signatures have identified distinct breast cancer subtypes associated with clinically-relevant disease outcomes [9], [20], [21]. In the present study, we have employed a genomic approach to facilitate the exploration of the biologic forces driving age-specific differences unique to breast cancer. Although unique clusters of pathway deregulation are representative of distinct phenotypes of breast cancer survival, the purpose of our analysis was not to generate yet another prognostic strategy. Instead, our goal was to describe an approach that could potentially explain the age-specific biologic differences seen in women with breast cancer, while also highlighting the potential for using targeted agents in a more rational manner–guided by the knowledge of oncogenic pathway deregulation.

Building on the expertise of applying oncogenic pathway deregulation, our analysis identified individual subsets of young women with prognostic differences defined by signatures of signaling pathway deregulation. Importantly, this analysis has allowed for the definition of a poorer prognosis subset of young women's breast cancer defined by a low probability of Src and E2F deregulation. Interestingly, this observation is congruent with previous reports illustrating poorer survival among patients across all ages with breast tumors characterized by lower than average E2F deregulation [9].

Although mutations in several E2F genes have been detected in many human cancers, results have been paradoxical [22]. High levels of E2F have been correlated with poorer outcome in several solid tumors [23], [24]. Conversely, reduced expression has been associated with aggressive disease suggesting a potential tumor suppressor role for E2F [25], [26]. Furthermore, inactivation of E2F was found to significantly accelerate tumor development in transgenic mice expressing Myc. This report parallels our observations and provides additional insight into specific oncogenic alterations cooperating with the loss of E2F [27]. It is postulated that the low probability of E2F pathway deregulation, in the context of PI3K, Myc and β-catenin pathway deregulation, is promoting tumorigenesis in this poor prognosis subset of young women with breast cancer.

Similar to E2F, the Src family kinases have been shown to contribute to the growth and survival of breast cancer cells [28]. It has also been observed that breast tumors expressing the progesterone receptor have higher observed Src activity [29]. Moreover, tumors arising in younger women are less likely to express either both estrogen and progesterone receptors–an observation that confers a poorer overall prognosis [3], [6]. The reported positive correlation between Src activity and hormone receptor status provides a potential explanation for the low probability of Src pathway, in the context of PI3K, Myc and β-catenin pathway deregulation, among the poor prognosis subset of young women in our analysis.

Of perhaps most importance is the potential for this data to reveal new therapeutic opportunities for patients at highest risk for breast cancer recurrence. Our past work has demonstrated an association between predicting pathway deregulation and sensitivity to therapeutics that target a component of the deregulated pathway[9]. The very poor prognosis group of young women with breast cancer is characterized by deregulation of PI3K, Myc, and β-catenin pathways. Of these, PI3K-specific therapies are available and represent a potential strategy that might be applied for this group of patients. Identification of unique subsets of patients by prognosis based on oncogenic pathway signatures provides not only an opportunity to tailor therapeutic approaches by recurrence risk, but also to incorporate targeted therapies geared toward individualized tumor biology with the ultimate goal of improving patient outcome.

## Supporting Information

Figure S1 Receiver operator curve (ROC) curve. ROC curve evaluating age and ER (IHC or EIA) status based on observations that breast cancer arising in younger women is less likely to express ER. Within this dataset, age less than approximately 45 years confers ER negativity (72% sensitivity; 53% specificity).
(0.42 MB TIF)

Figure S2 Oncogenic pathway deregulation among human breast tumors by ER status among women aged ≤45 years. A) LEFT PANEL. Hierarchical clustering of ER positive human breast tumors. Two main clusters emerge: low probability of Src deregulation (cluster 1) and high probability of Src deregulation (cluster 2); RIGHT PANEL. Kaplan Meier survival analysis for patient with ER positive breast cancer based on Src pathway deregulation. B) LEFT PANEL Hierarchical clustering of ER negative human breast tumors. Two main clusters emerge: low probability of Src deregulation (cluster 1) and high probability of Src deregulation (cluster 2); RIGHT PANEL Kaplan Meier survival analysis for patients with ER negative breast cancer based on Src pathway deregulation.
(0.92 MB TIF)

Figure S3 Pathway deregulation among human breast tumors arising in women aged ≤45 years by lymph node status. A) LEFT PANEL Hierarchical clustering of predictions of pathway deregulation among lymph node positive human breast tumors; RIGHT PANEL Kaplan Meier survival analysis for young, lymph node positive breast cancer patients based on Src pathway deregulation. B) LEFT PANEL Hierarchical clustering of predictions of pathway deregulation among lymph node negative human breast tumors; RIGHT PANEL Kaplan Meier survival analysis for young, lymph node negative breast cancer patients based on PI3K pathway deregulation.
(0.93 MB TIF)

Figure S4 Pathway deregulation among human breast tumors arising in women aged ≤45 years by Her2 status. A) LEFT PANEL Hierarchical clustering of predictions of pathway deregulation among Her2 0-1+ (IHC) human breast tumors; RIGHT PANEL Kaplan Meier survival analysis for young, Her2 0-1+ (IHC) breast cancer patients based on PI3K pathway deregulation. B) LEFT PANEL Hierarchical clustering of predictions of pathway deregulation among Her2 2-3+ (IHC) human breast tumors; RIGHT PANEL Kaplan Meier survival analysis for young, Her2 2-3+ (IHC) breast cancer patients based on PI3K pathway deregulation.
(0.65 MB TIF)

Figure S5 Pathway deregulation among human breast tumors arising in women aged ≤45 years by grade. A) LEFT PANEL Hierarchical clustering of predictions of pathway deregulation among grade 1–2 human breast tumors; RIGHT PANEL Kaplan Meier survival analysis for young, grade 1–2 breast cancer patients based on Src pathway deregulation. B ) LEFT PANEL Hierarchical clustering of predictions of pathway deregulation among grade 3 human breast tumors; RIGHT PANEL Kaplan Meier survival analysis for young, grade 3 breast cancer patients based on PI3K pathway deregulation.
(0.80 MB TIF)

Figure S6 Pathway deregulation among human breast tumors arising women aged ≤45 years by tumor size (T≤2 cm vs. T>2 cm). A) LEFT PANEL. Hierarchical clustering of predictions of pathway deregulation in samples of human breast tumors ≤2 cm; RIGHT PANEL Kaplan Meier survival analysis for young women with breast tumors ≤2 cm based on Src pathway deregulation. B) LEFT PANEL. Hierarchical clustering of predictions of pathway deregulation in samples of human breast tumors >2 cm; RIGHT PANEL Kaplan Meier survival analysis for young women with breast tumors >2 cm based on Src pathway deregulation.
(0.82 MB TIF)

Table S1 Dataset Details
(0.02 MB DOC)

Table S2 Clinical Characteristics by Age (≤45 yrs, ≥65 yrs)
(0.03 MB DOC)

Methods S1 Supplementary Methods
(0.03 MB DOC)
