# Supplementary figures and images for: Age-Specific Differences in Oncogenic Pathway Deregulation Seen in Human Breast Tumors
Source: PLoS One. 2008 Jan 2;3(1):e1373. doi: 10.1371/journal.pone.0001373 (PMC2148101; doi:10.1371/journal.pone.0001373)

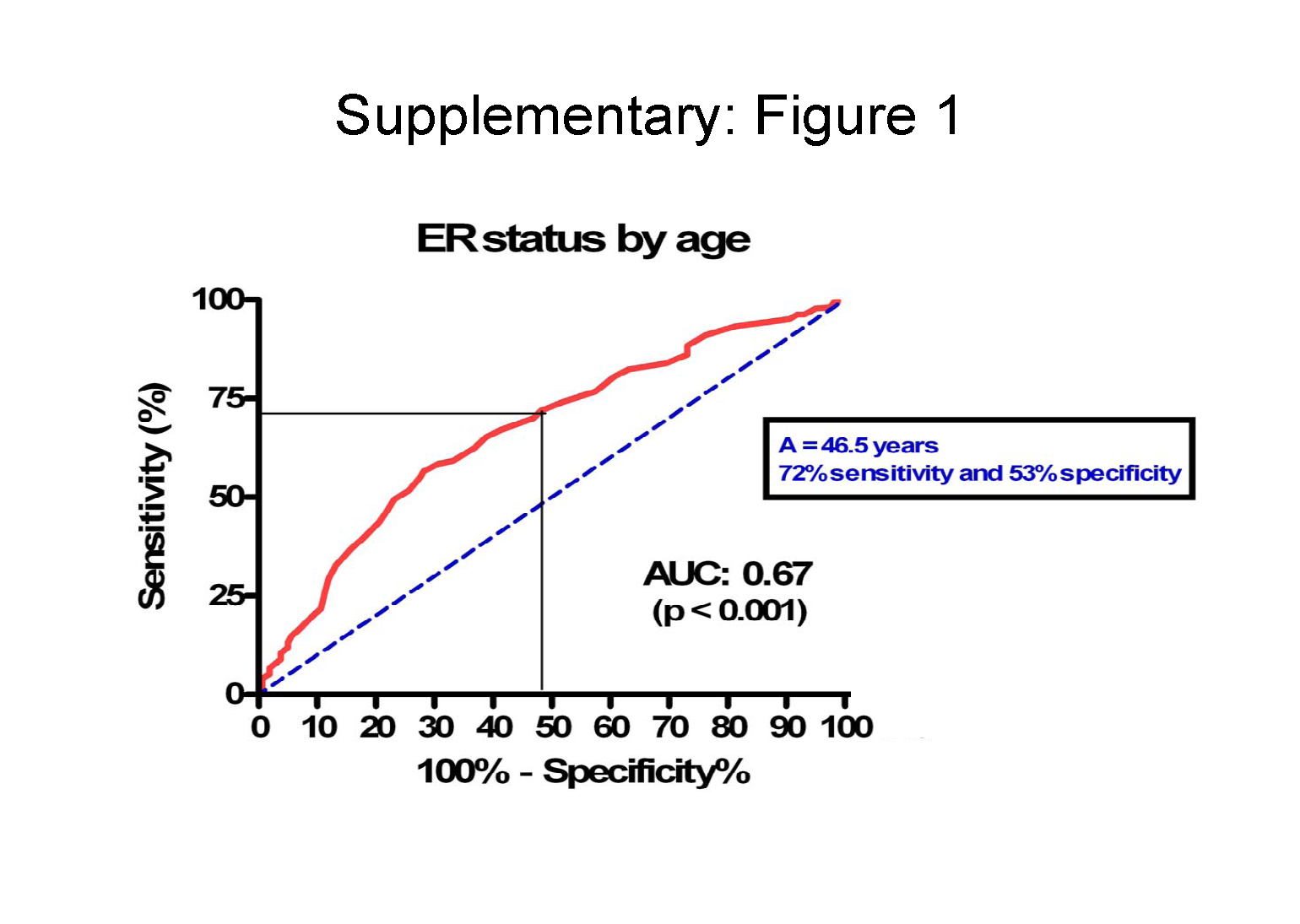

Supplement: Figure S1 — Receiver operator curve (ROC) curve. ROC curve evaluating age and ER (IHC or EIA) status based on observations that breast cancer arising in younger women is less likely to express ER. Within this dataset, age less than approximately 45 years confers ER negativity (72% sensitivity; 53% specificity). (0.42 MB TIF) [file pone.0001373.s001.tif]

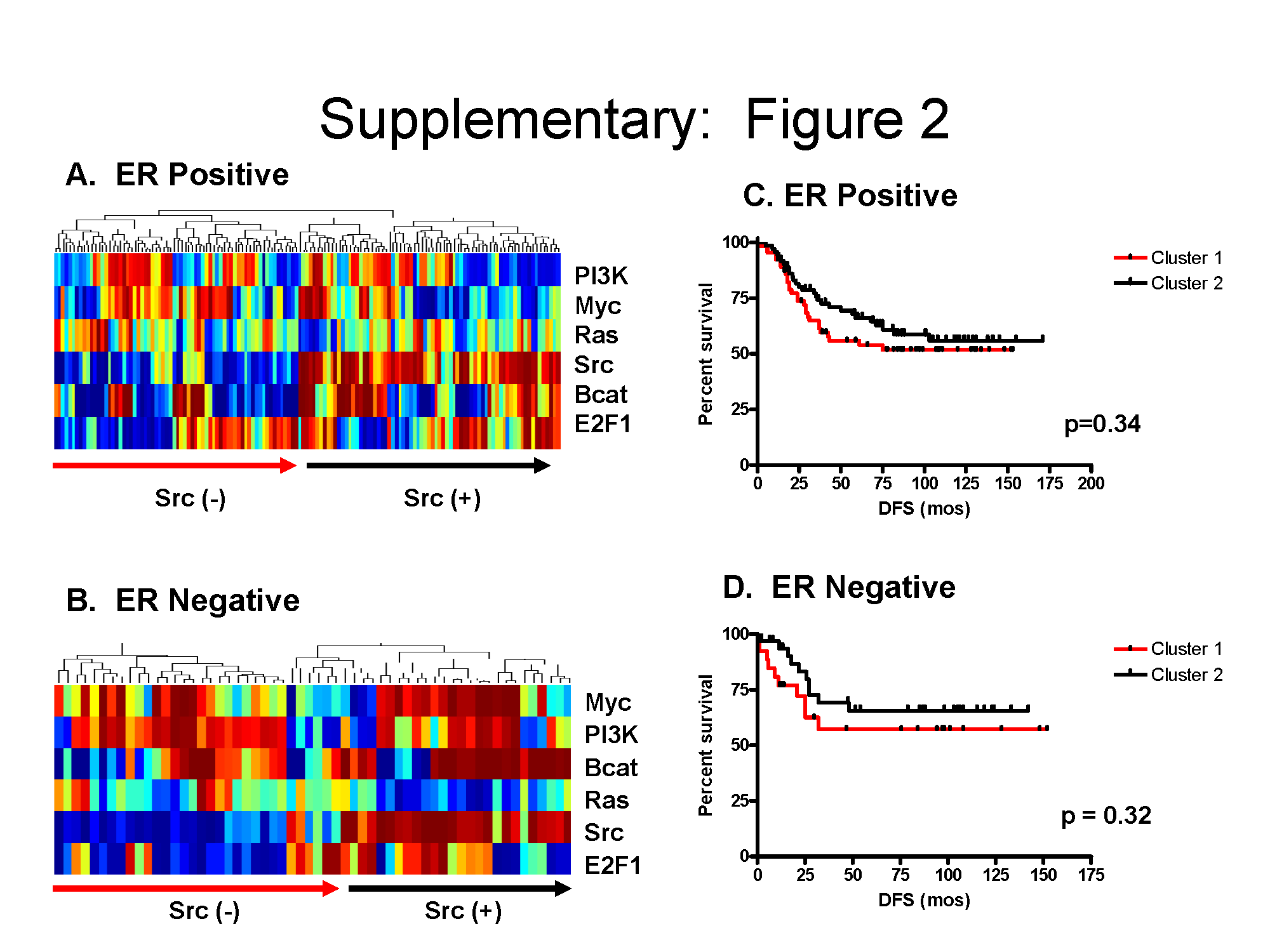

Supplement: Figure S2 — Oncogenic pathway deregulation among human breast tumors by ER status among women aged ≤45 years. A) LEFT PANEL. Hierarchical clustering of ER positive human breast tumors. Two main clusters emerge: low probability of Src deregulation (cluster 1) and high probability of Src deregulation (cluster 2); RIGHT PANEL. Kaplan Meier survival analysis for patient with ER positive breast cancer based on Src pathway deregulation. B) LEFT PANEL Hierarchical clustering of ER negative human breast tumors. Two main clusters emerge: low probability of Src deregulation (cluster 1) and high probability of Src deregulation (cluster 2); RIGHT PANEL Kaplan Meier survival analysis for patients with ER negative breast cancer based on Src pathway deregulation. (0.92 MB TIF) [file pone.0001373.s002.tif]

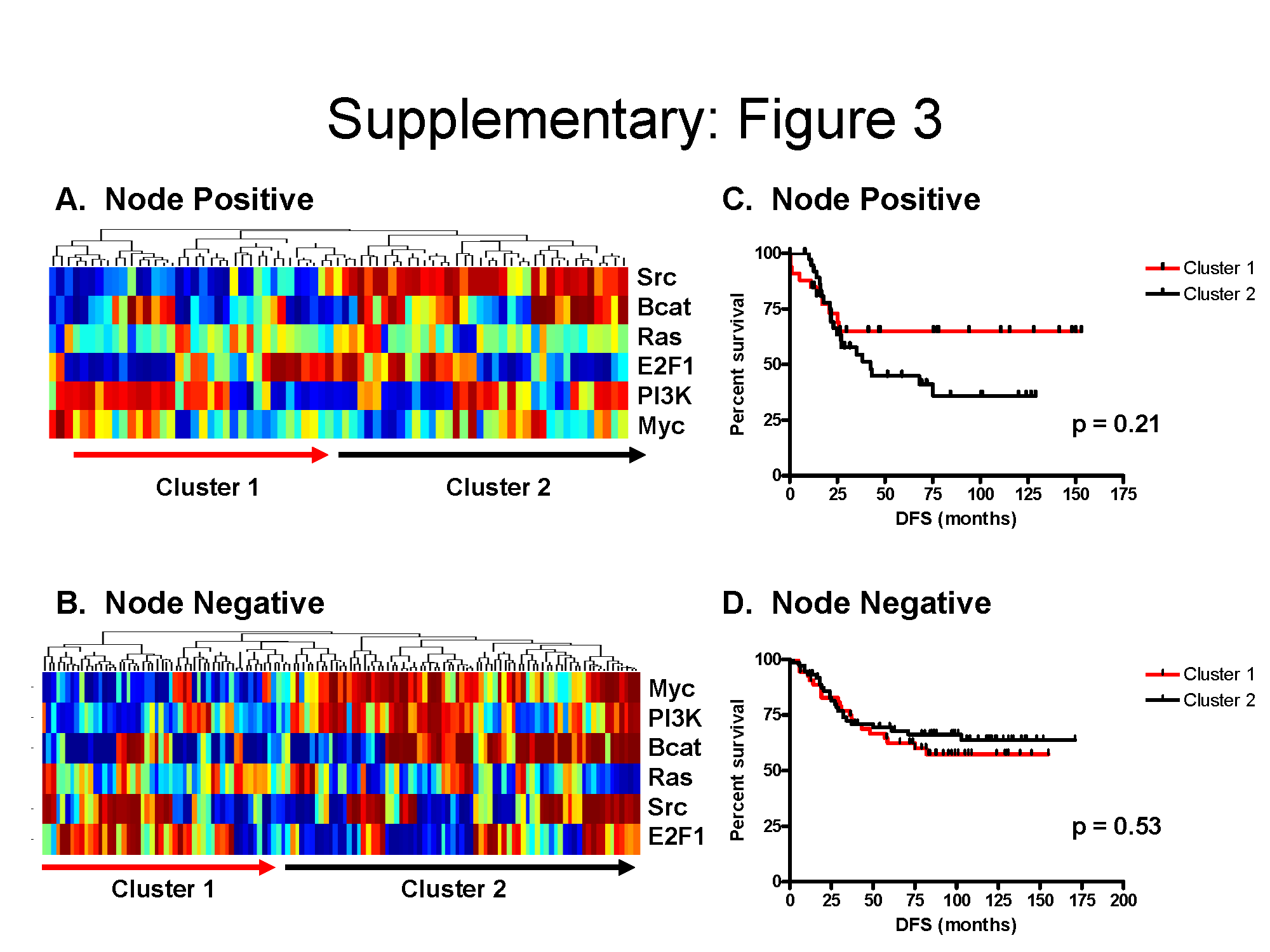

Supplement: Figure S3 — Pathway deregulation among human breast tumors arising in women aged ≤45 years by lymph node status. A) LEFT PANEL Hierarchical clustering of predictions of pathway deregulation among lymph node positive human breast tumors; RIGHT PANEL Kaplan Meier survival analysis for young, lymph node positive breast cancer patients based on Src pathway deregulation. B) LEFT PANEL Hierarchical clustering of predictions of pathway deregulation among lymph node negative human breast tumors; RIGHT PANEL Kaplan Meier survival analysis for young, lymph node negative breast cancer patients based on PI3K pathway deregulation. (0.93 MB TIF) [file pone.0001373.s003.tif]

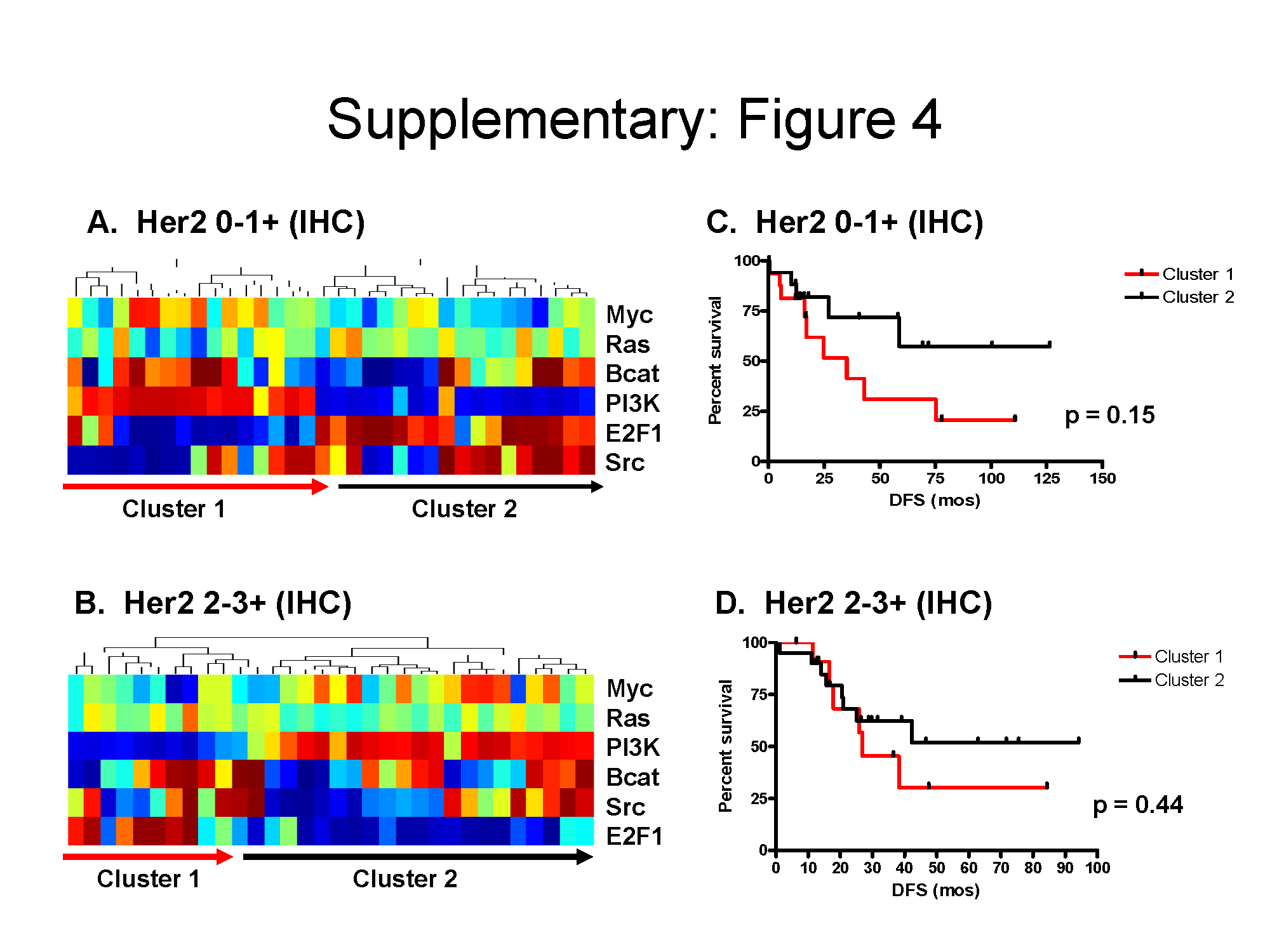

Supplement: Figure S4 — Pathway deregulation among human breast tumors arising in women aged ≤45 years by Her2 status. A) LEFT PANEL Hierarchical clustering of predictions of pathway deregulation among Her2 0-1+ (IHC) human breast tumors; RIGHT PANEL Kaplan Meier survival analysis for young, Her2 0-1+ (IHC) breast cancer patients based on PI3K pathway deregulation. B) LEFT PANEL Hierarchical clustering of predictions of pathway deregulation among Her2 2-3+ (IHC) human breast tumors; RIGHT PANEL Kaplan Meier survival analysis for young, Her2 2-3+ (IHC) breast cancer patients based on PI3K pathway deregulation. (0.65 MB TIF) [file pone.0001373.s004.tif]

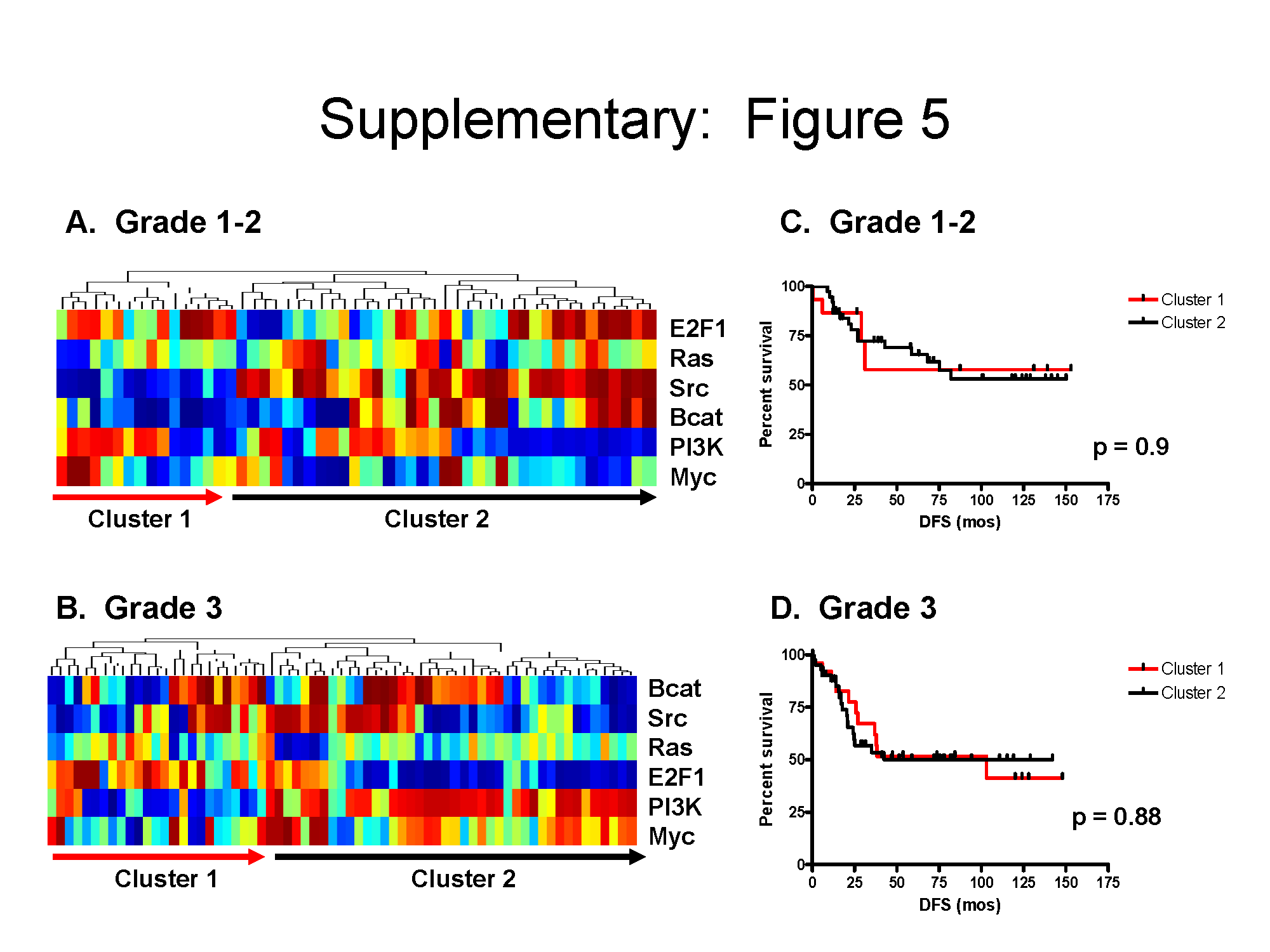

Supplement: Figure S5 — Pathway deregulation among human breast tumors arising in women aged ≤45 years by grade. A) LEFT PANEL Hierarchical clustering of predictions of pathway deregulation among grade 1–2 human breast tumors; RIGHT PANEL Kaplan Meier survival analysis for young, grade 1–2 breast cancer patients based on Src pathway deregulation. B ) LEFT PANEL Hierarchical clustering of predictions of pathway deregulation among grade 3 human breast tumors; RIGHT PANEL Kaplan Meier survival analysis for young, grade 3 breast cancer patients based on PI3K pathway deregulation. (0.80 MB TIF) [file pone.0001373.s005.tif]

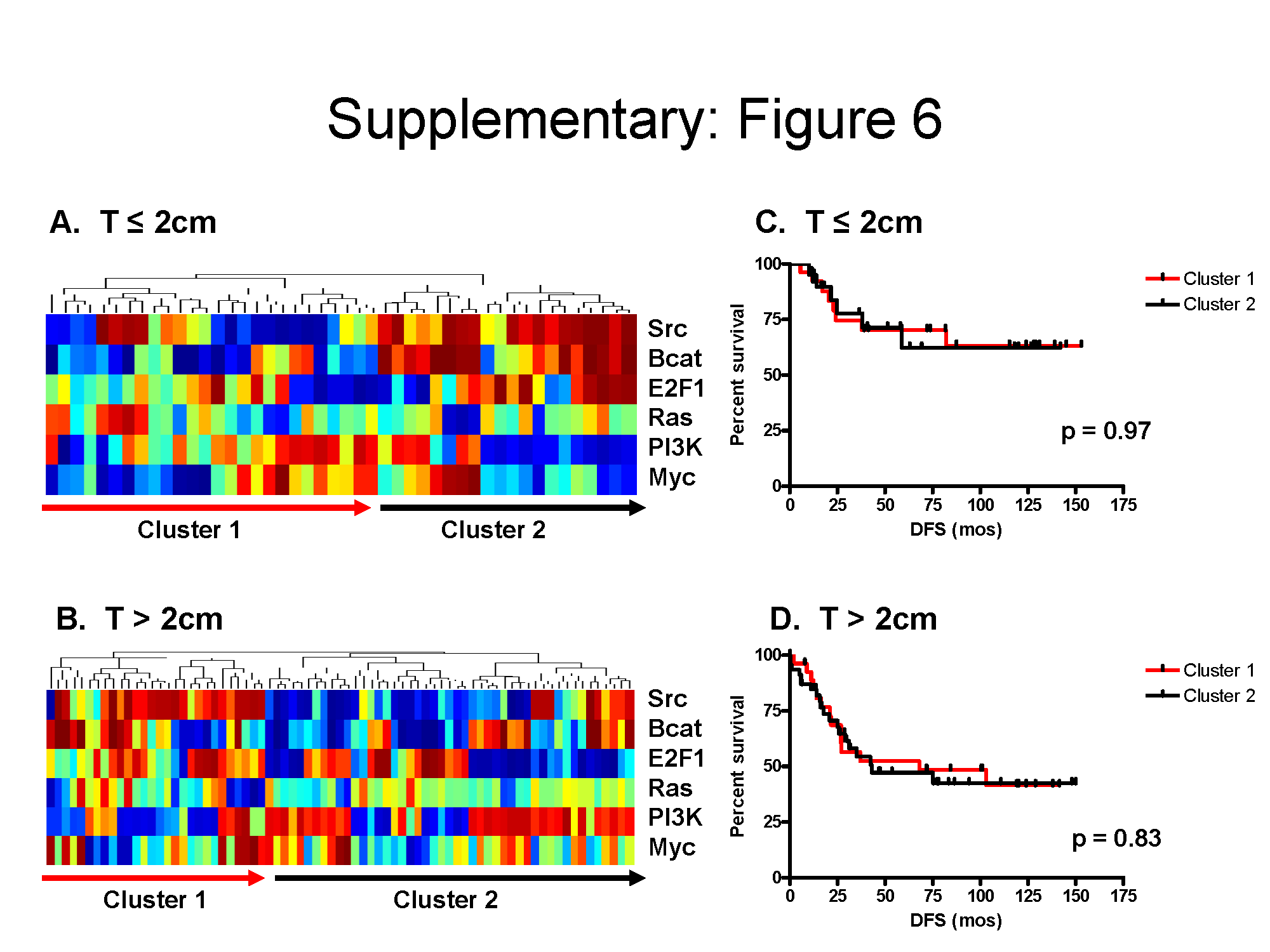

Supplement: Figure S6 — Pathway deregulation among human breast tumors arising women aged ≤45 years by tumor size (T≤2 cm vs. T>2 cm). A) LEFT PANEL. Hierarchical clustering of predictions of pathway deregulation in samples of human breast tumors ≤2 cm; RIGHT PANEL Kaplan Meier survival analysis for young women with breast tumors ≤2 cm based on Src pathway deregulation. B) LEFT PANEL. Hierarchical clustering of predictions of pathway deregulation in samples of human breast tumors >2 cm; RIGHT PANEL Kaplan Meier survival analysis for young women with breast tumors >2 cm based on Src pathway deregulation. (0.82 MB TIF) [file pone.0001373.s006.tif]
