# Supplementary material for: Age-Specific Differences in Oncogenic Pathway Deregulation Seen in Human Breast Tumors
Source: PLoS One. 2008 Jan 2;3(1):e1373. doi: 10.1371/journal.pone.0001373 (PMC2148101; doi:10.1371/journal.pone.0001373)
Supplement: Table S1 — Dataset Details (0.02 MB DOC) [file pone.0001373.s007.doc]

**Supplementary Table S1: Dataset Details**

**Duke Codex GSE 2034 GSE 4922**

**Total (n): n = 78 n = 171 n = 286 n =289**

**Dates: 1990-2001 1991-2000 1980-1995 1987-2002**

**Geographic**

**Regions: United States Taiwan Netherlands Sweden**

**Singapore***

**Affymetrix**

**Platform: U95 U95 U133A U133A**

**Endpoint DDFS DFS DDFS DFS**

**Detection Method:**

**ER/PR IHC IHC EIA, IHC EIA**

**Her2 n/a IHC n/a n/a**

Key: EIA = enzyme immunoassay, IHC = immunohistochemistry, DFS = disease free survival, DDFS = distant disease free survival

*Clinical variables were unavailable for Singapore samples (n=40).
