# Supplementary material for: Age-Specific Differences in Oncogenic Pathway Deregulation Seen in Human Breast Tumors
Source: PLoS One. 2008 Jan 2;3(1):e1373. doi: 10.1371/journal.pone.0001373 (PMC2148101; doi:10.1371/journal.pone.0001373)
Supplement: Table S2 — Clinical Characteristics by Age (≤45 yrs, ≥65 yrs) (0.03 MB DOC) [file pone.0001373.s008.doc]

**Supplementary Table S2: Clinical Characteristics by Age (≤ 45 yrs, ≥ 65 yrs)**

**Duke Codex GSE 2034 GSE 4922**

**≤ 45 y ≥ 65 y ≤ 45 y ≥ 65 y ≤ 45 y ≥ 65 y ≤ 45 y ≥ 65 y**

n=11 n=10 n=76 n=22 n=79 n=57 n=34 n=122

Characterisitic

No. (%)

Median Age, yrs 41 67 39 67.5 41 70 40 73

Range, yrs 33-45 65-77 27-45 65-77 26-45 65-83 28-45 65-93

Race ─ ─ ─ ─ ─ ─

White 9 (82) 8 (80)

Black 2 (18) 2 (20)

ER status

Positive 9 (82) 6 (60) 24 (32) 16 (73) 52 (66) 42 (74) 27(79) 102 (83)

Negative 1 (9) 3 (30) 52 (68) 6(27) 27 (34) 15 (26) 6 (18) 17(14)

Missing 1 (9) 1 (10) 0 0 0 0 1 (3) 3(3)

PR status ─ ─ ─ ─ ─ ─

Positive 6 (55) 5 (50)

Negative 2 (18) 5 (50)

Missing 3 (27) 0

Her2 status ─ ─ ─ ─ ─ ─

0-1+ 34 (45) 16 (73)

2-3+ 32 (42) 5(23)

Missing 10 (13) 1(4)

Lympn Node Status

Positive 4 (36) 6 (60) 57 (75) 15 (68) 0 0 12 (35) 30 (25)

Negative 7 (64) 4 (40) 19 (25) 7 (32) 79 (100) 57 (100) 22(65) 83 68) Missing 0 0 0 0 0 0 0 9 (7)

Tumor Size

Median, cm 1.0 2.3 2.6 3.0 2.2 2.2 2.2 1.9

Range, cm 0.8-4.5 0.8-4.5 0.2-8.5 0.7-11.5 1.0-8.0 0.5-5.0 1.1-13 0.2-6.5

Missing 0 0 0 0 9 (11) 0 0 0

Adjuvant chemotherapy* ─ ─

Yes 7 (64) 2 (20) 66 (87) 10 (45) 0 0

No 4 (36) 8 (80) 10 (13) 12 (55) 79(100) 57 (100)

*Includes anthracyclines and taxane-based therapies.
