# Supplementary material for: Age-Specific Differences in Oncogenic Pathway Deregulation Seen in Human Breast Tumors
Source: PLoS One. 2008 Jan 2;3(1):e1373. doi: 10.1371/journal.pone.0001373 (PMC2148101; doi:10.1371/journal.pone.0001373)
Supplement: Methods S1 — Supplementary Methods (0.03 MB DOC) [file pone.0001373.s009.doc]

**Supplementary Methods:**

**Statistical Analysis Methods**

Prior to statistical modeling, gene expression data is filtered to exclude probesets with signals present at background noise levels, and for probesets that do not vary significantly across samples. A metagene represents a group of genes that together exhibit a consistent pattern of expression in relation to an observable phenotype. Each signature summarizes its constituent genes as a single expression profile, and is here derived as the first principal component of that set of genes (the factor corresponding to the largest singular value) as determined by a singular value decomposition. Given a training set of expression vectors (of values across metagenes) representing two biological states, a binary probit regression model is estimated using Bayesian methods. Applied to a separate validation data set, this leads to evaluations of predictive probabilities of each of the two states for each case in the validation set.

When predicting the pathway activation or chemosensitivity patterns of cancer cell lines or tumor samples, gene selection and identification is based on the training data, and then metagene values are computed using the principal components of the training data and additional cell line or tumor expression data. Bayesian fitting of binary probit regression models to the training data then permits an assessment of the relevance of the metagene signatures in within-sample classification, and estimation and uncertainty assessments for the binary regression weights mapping metagenes to probabilities of relative pathway status or chemosenstivity patterns. Predictions of the relative pathway status or chemosensitivity patterns of the validation cell lines or tumor samples are then evaluated, producing estimated relative probabilities – and associated measures of uncertainty – of activation/deregulation across the validation samples.

**Cross-platform Affymetrix Gene Chip comparison.** To map the probe sets across various generations of Affymetrix GeneChip arrays, we utilized an in-house program, Chip Comparer (<http://tenero.duhs.duke.edu/genearray/perl/chip/chipcomparer.pl>). First, each probeset ID in given Affymetrix gene chips were mapped to the corresponding LocusID. This is done by parsing local copies of LocusLink and UniGene databases to identify inherent relationship between the GenBank accession number associated with each probeset sequence and its corresponding LocusID. Second, probesets from different gene chips are matched by sharing the same LocusID (or orthologous pair of LocusIDs in the case of mapping gene chips across species).
